# A case report of secondary retroperitoneal lymph node tuberculosis following chemotherapy for acute myeloid leukemia with literature review

**DOI:** 10.3389/fonc.2026.1792938

**Published:** 2026-04-20

**Authors:** Yizhuo Liu, Yuxia Jiang, Fenglin Shen, Mingxia He, Yunfeng Sun, Xiaofeng Xu, Huifang Jiang

**Affiliations:** 1 Tongde Hospital of Zhejiang Province Affiliated to Zhejiang Chinese Medical University (College of Integrated Traditional Chinese and Western Medicine Clinical Medicine), Hangzhou, Zhangjiang, China; 2 Department of Hematology, Tongde Hospital of Zhejiang Province, Hangzhou, Zhangjiang, China; 3 Department of Oncology and Hematology, Hangzhou Red Cross Hospital, Hangzhou, Zhejiang, China

**Keywords:** acute myeloid leukemia, antituberculosis therapy, immunosuppression, lymphnode puncture, retroperitoneal lymph node tuberculosis, T-spot test

## Abstract

The incidence of secondary retroperitoneal lymph node tuberculosis (TB) in patients with acute myeloid leukemia (AML) is relatively low, making it challenging to diagnose in clinical practice. This paper reports an AML patient who developed TB after chemotherapy, confirmed by positive T-SPOT results from the ultrasound-guided retroperitoneal lymph node biopsy. Eventually, the patient achieved negative conversion on T-SPOT test following individualized anti-TB treatment. This case highlights the necessity of the diagnosis of tuberculosis when infections occur in leukemia patients. Clinically, ultrasound-guided biopsy enables timely diagnosis, and favorable outcome can be attained by selecting an appropriate therapeutic regimen.

## Introduction

Acute myeloid leukemia (AML) is a clonal disorder characterized by the uncontrolled proliferation of hematopoietic cells, frequently accompanied by infection, anemia, and hemorrhage (1). The primary therapies for AML include chemotherapy, hematopoietic stem cell transplantation(HSCT), and targeted therapies. Currently, intensive chemotherapy is recommended for AML patients who can tolerate such regimens (2). And for elderly AML patients or any other patients who are intolerant to intensive chemotherapy, the ASH Guidelines Panel recommends the hypomethylating agent monotherapy or low-dose cytarabine monotherapy (3). Tuberculosis (TB) is an infectious disease caused by Mycobacterium tuberculosis, and about one-quarter of the global population is estimated to be infected with the pathogen (4). Although TB is relatively common in China, fever and other related symptoms are also frequently observed in AML (5). Atypical clinical presentations make TB prone to misdiagnosis and underdiagnosis in the patients with AML. Furthermore, the extremely limited number of clinical reports on secondary retroperitoneal lymph node tuberculosis makes it more likely to lead to missed diagnosis or misdiagnosis in AML patients. Thus, we report an AML patient complicated by tuberculosis after chemotherapy to improve the diagnosis and treatment.

## Case description

A 74-year-old man underwent a routine blood examination at another hospital during treatment for lung cancer, which revealed a white blood cell (WBC) count of 67.8×10^9^/L and a platelet count of 19×10^9^/L. He was then diagnosed with acute myeloid leukemia, FAB subtype M2. Molecular analysis revealed the presence of a NUP98::TOP1 fusion gene, along with mutations in FLT3, WT1, CEBPA, and PTPN11. Cytogenetic analysis demonstrated a t (11, 20)(p15;q11.2) translocation. This genetic profile corresponded to the WHO classification of acute myeloid leukemia with NUP98 rearrangement and initiated oral hydroxyurea at a dose of 0.5 g twice daily at another hospital on January 10, 2025. On January 13, 2025, the patient began induction chemotherapy with cytarabine 136 mg on days 1–7, venetoclax 100 mg on day 2, 200 mg on day 3, and 400 mg on days 4–10. Then he was admitted to our department (the Department of Hematology, Tongde Hospital of Zhejiang Province) on January 27, 2025. Bone marrow aspiration revealed that blasts accounted for 15% of the total nucleated cells with the flow cytometry identifying 25.18% abnormal early myeloid cells. On February 12, 2025, the patient initiated chemotherapy with azacitidine (75 mg daily) for 7 consecutive days, in combination with oral venetoclax (200 mg once daily) and alternating doses of oral gilteritinib (40 mg or 80 mg once daily) for 14 consecutive days. A subsequent bone marrow aspiration for disease reassessment was performed on March 5, 2025, which revealed approximately 3% myeloblasts on the bone marrow smear and 1.82% residual tumor cells on the flow cytometry. On March 6, the patient continued maintenance chemotherapy with alternating doses of oral gilteritinib (40 mg or 80 mg once daily).

**Figure 1:**
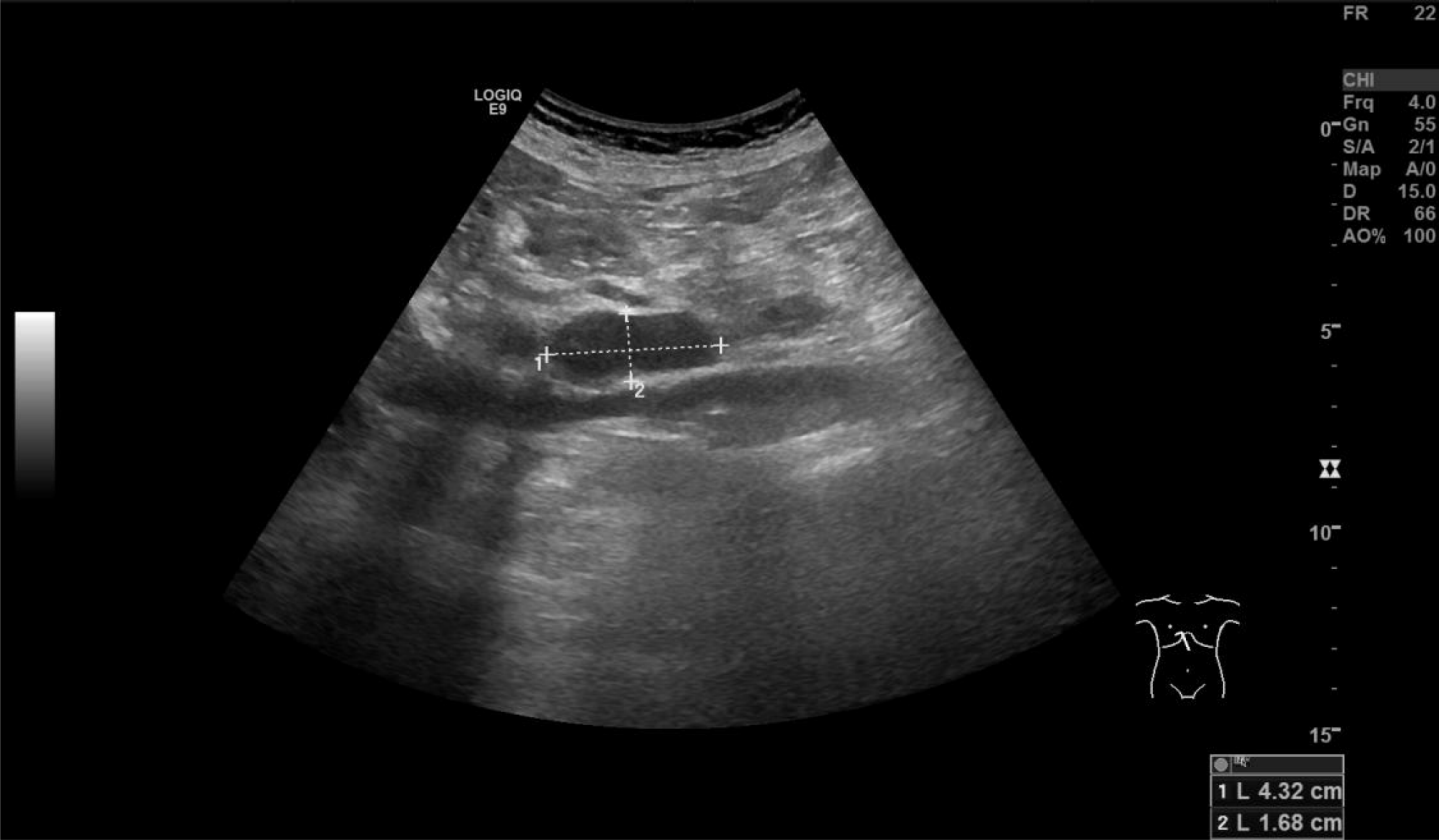
Retroperitoneal lymph node ultrasonography for the first time, the largest mase was measured at approximately 4.0×2.0 cm.

**Figure 2:**
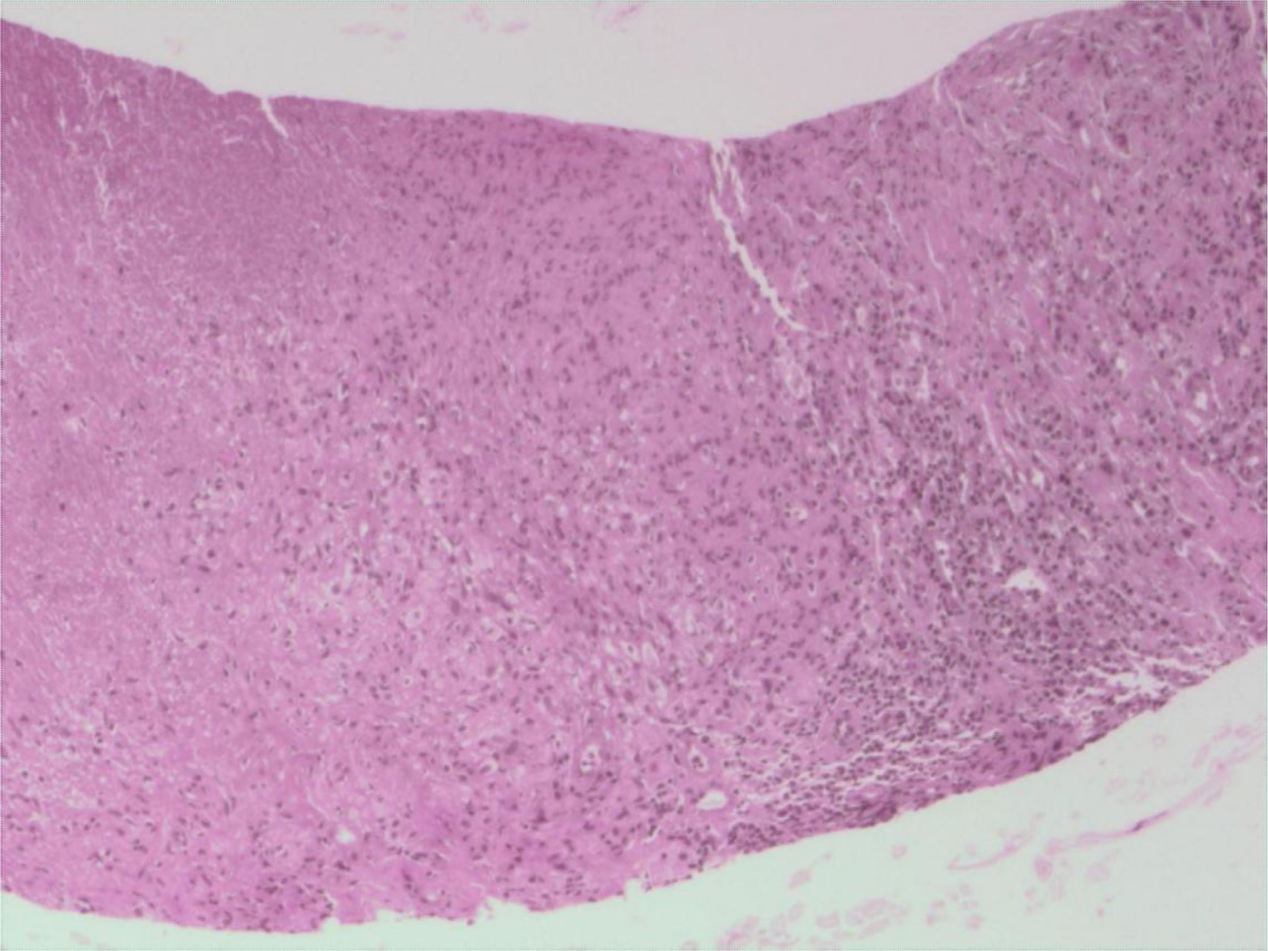
Pathological examination of the retroperitoneal lymph node revealed granulomatous inflammation with necrosis.

**Figure 3:**
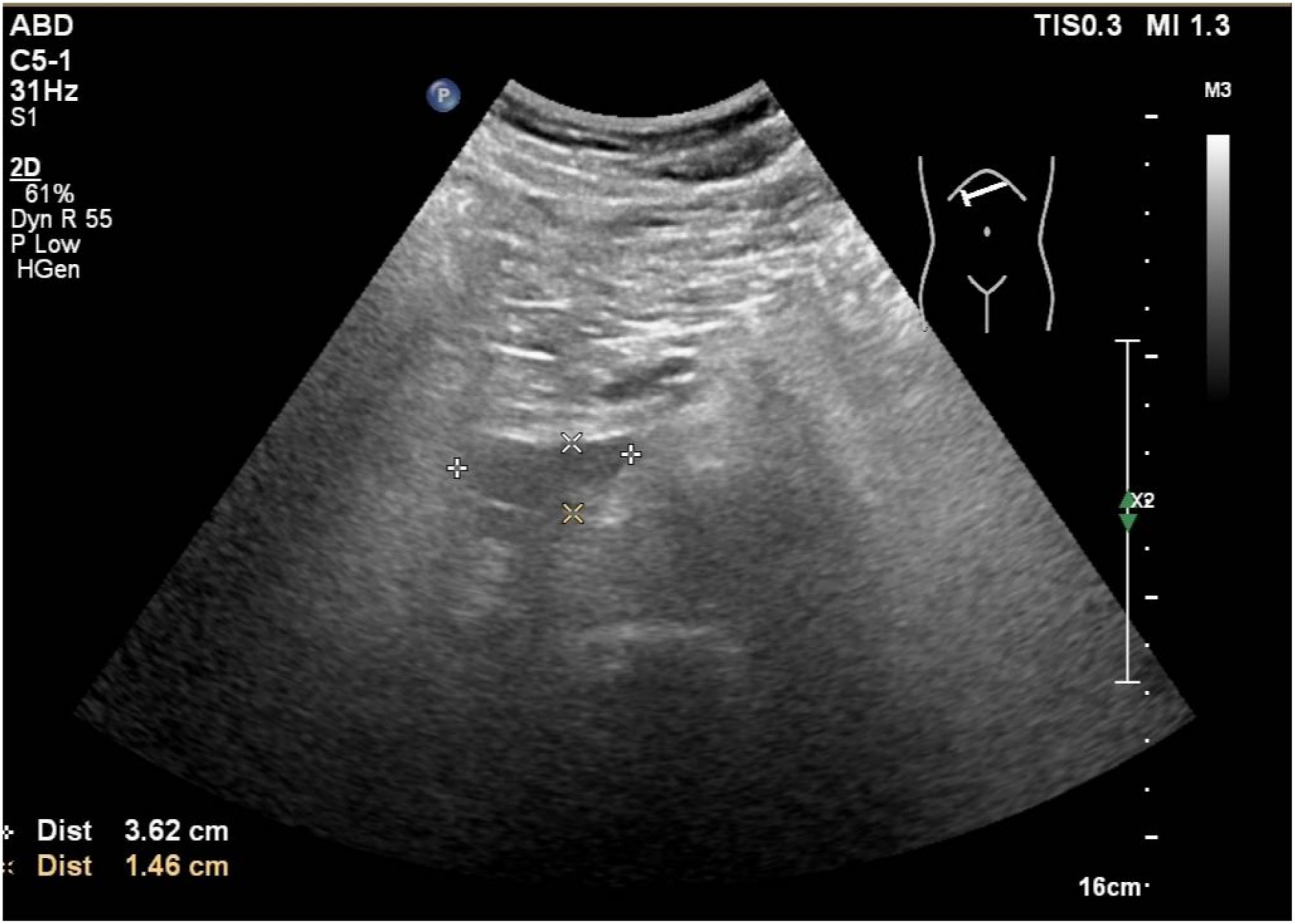
Follow-up ultrasonography of abdominal retroperitoneal lymph nodes after anti-tuberculosis Treatment, and the lymph node mase had a reduction to 3.6×1.5 cm.

During the treatment, the patient developed a cough with expectoration, complicated by febrile neutropenia (absolute neutrophil count: 0.1×10^9^/L) and an elevated C-reactive protein (CRP) level of 21.3 mg/L. A chest computed tomography (CT) revealed pulmonary infection. The patient received sequential intravenous antibiotic therapy including moxifloxacin, meropenem, omadacycline, and voriconazole. However, the treatment showed a limited efficacy, and the patient continued to experience recurrent fever. On March 18, 2025, thoracentesis with drainage was performed. The pleural effusion was hemorrhagic. Flow cytometry analysis of the effusion revealed no phenotypically distinct abnormal cell populations, and pathological examination of exfoliated cells showed no definitive evidence of malignancy. A follow-up ultrasound of superficial lymph nodes on March 22 revealed multiple retroperitoneal lymph node masses, the largest one was measured at approximately 4.0×2.0 cm (**Figure 1**). On March 27, an ultrasound-guided percutaneous needle biopsy of the retroperitoneal masses was performed. Pathological examination revealed granulomatous inflammation with necrosis, acid-fast bacilli were detected (**Figure 2**), and the T-SPOT test had a positive result, whereas the mycobacterial testing of sputum samples was negative. Based on these findings, the patient was diagnosed with retroperitoneal lymph node tuberculosis. On March 31, 2025, a repeat bone marrow aspiration revealed no detectable blasts on smear examination, consistent with leukemia remission, and the flow cytometry indicated residual tumor cells at 0.23%, confirming the achievement of complete remission (CR). Subsequently, the patient continued to receive low-intensity chemotherapy with venetoclax combined with gilteritinib. Meanwhile, he initiated a combined anti-tuberculosis therapy consisting of isoniazid, ethambutol, and moxifloxacin on April 3, 2025. Serial bone marrow aspiration confirmed sustained bone marrow remission (**Table 1**). A follow-up retroperitoneal ultrasound on June 17, 2025, demonstrated a reduction in the lymph node masses to 3.6×1.5 cm (**Figure 3**). On June 18, 2025, a repeat T-SPOT test converted to negative for the first time. Meanwhile, the patient's blood counts gradually improved, and no significant abnormalities in liver or kidney function were observed throughout the course of treatment (**Table 2**).

**Table 1:** The results of bone marrow puncture throughout the course of the disease.

Date	Myeloblasts (%)	Result
3/11	3	MRD
4/1	0.5	MRD
5/12	1.5	MRD
6/17	0	MRD
7/21	2	MRD

**Table 2:** Timeline of relevant blood routine and biochemical indicators during the patient’s illness course.

Date	ANC (*10^9^/L)	HBC (g/L)	PLT (*10^9^/L)	ALT (U/L)	AST (U/L)	Cr (μmol/L)	Treantment
3/19	0.2	74	89	20	n/a	67	NO
3/22	1.2	61	89	26	n/a	49	NO
3/27	1.4	65	93	23	n/a	57	NO
3/29	1.2	67	89	19	n/a	59	NO
4/1	0.5	67	128	27	n/a	53	NO
4/5	2.5	65	124	46	n/a	55	YES
4/7	2.6	60	136	31	20	56	YES
4/13	1.1	77	188	33	24	56	YES
4/20	1.4	92	164	47	n/a	67	YES
4/26	2.2	99	136	49	27	57	YES
5/3	1.3	100	122	30	24	66	YES
5/11	5.5	104	156	24	n/a	56	YES
5/18	3.7	112	164	22	26	62	YES
5/25	1.2	110	153	31	28	66	YES
6/2	1.0	111	145	28	n/a	72	YES
6/16	1.2	123	164	23	28	71	YES
6/19	1.9	106	151	21	26	56	YES

ANC, Absolute Neutrophil Count; HBC, Hemoglobin count; PLT, platelet count; ALT, Alanine Aminotransferase; AST, Aspartate Aminotransferase; Cr, Creatinine.

## Discussion

Acute myeloid leukemia (AML) is a malignant myeloid disorder characterized by clonal expansion and impaired differentiation of myeloid progenitor cells (6). Patients with AML are frequently immunocompromised due to the disease itself. Additionally, the effects of chemotherapy often result in leukopenia and neutropenia, further compromising host defenses and making them susceptible to concurrent infections. Decreased CD4+ T-cell counts, impaired CD8+ T-cell function, and a reduced CD4+/CD8+ ratio in leukemia patients, have also been implicated as potential mechanisms underlying the increased susceptibility to tuberculosis (7). Clinically, the risk of tuberculosis in patients with malignant hematological tumors is approximately 9 times higher than that in the general population, with leukemia being the most frequent among these malignancies (8) (9). Therefore, the prevention and treatment of tuberculosis are of particular importance in patients with leukemia. Studies have reported variable diagnostic performance of the T-SPOT test. One study demonstrated a positive rate of the T-SPOT test was 79.17%, with a diagnostic sensitivity of 91.84% and a specificity of 77.27%, suggesting its utility for early screening (10). Another study reported a sensitivity of the T-SPOT test was 81%, while a specificity was only 59%, indicating that a negative result may be more reliable for excluding tuberculosis than a positive result for confirming it (11) (12). Conversely, a retrospective study by Sun et al. concluded that the T-SPOT test not only can be a diagnostic tool for latent tuberculosis but also has an auxiliary diagnostic value for active tuberculosis (13).

Pathological examination and mycobacterial culture of lesions tissue are considered the gold standard for the diagnosis of lymph node tuberculosis (14). Traditionally, excisional lymph node biopsy has been the standard method for tissue sampling, as it provides sufficient tissue for comprehensive evaluation. However, this procedure requires surgical scheduling and is associated with considerable trauma. A retrospective study suggested that core needle biopsy of lymph nodes can reduce the need for surgery, which offers a more cost-effective approach and provides a faster diagnosis, making it a preferred first-line diagnostic modality (15). In summary, ultrasound-guided needle biopsy of lesions not only ensures accurate sampling of both superficial and deep lesions but also offers advantages including safety, high diagnostic yield, and diagnostic accuracy comparable to surgical biopsy (16).

There is currently no standardized treatment protocol for tuberculosis secondary to AML. Some studies have suggested that compared with the high mortality rate of tuberculosis in other hematological malignancies, tuberculosis occurring in acute leukemia tends to follow a more benign course and responds well to anti-tuberculosis therapy (9). Therefore, it is generally considered feasible to administer conventional chemotherapy for leukemia concurrently with anti-tuberculosis treatment. A case report described two AML patients complicated with tuberculosis who received conventional anti-tuberculosis treatment (2HREZ/4RH regimen: isoniazid, rifampicin, ethambutol, and pyrazinamide) in combination with cytarabine chemotherapy. Both achieved favorable outcomes (17). In another report, two patients were described: one patient received intensive anti-tuberculosis therapy with HRZE (isoniazid, rifampicin, ethambutol, and pyrazinamide) combined with a VHA chemotherapy regimen (venetoclax, homoharringtonine, and cytarabine), and the other received oral HRZE combined with a VA chemotherapy regimen (venetoclax and azacitidine). In subsequent visits, both patients had achieved complete remission of AML and effective control of active tuberculosis (18). Another case involved an AML patient harboring DNA methyltransferase 3A (DNMT3A), FMS-like tyrosine kinase 3-tyrosine kinase domain (FLT3-TKD), and Isocitrate dehydrogenase 2 (IDH2) mutations who develpoed active tuberculosis. The patient received anti-tuberculosis therapy (levofloxacin, isoniazid, pyrazinamide, and ethambutol) in combination with VHA chemotherapy, ultimately achieving significant disease remission, which facilitated subsequent hematopoietic stem cell transplantation. This suggests that timely anti-leukemia treatment is crucial for such patients on the premise of aggressive anti-tuberculosis therapy (19). A study suggested that anti-tuberculosis treatment is feasible in acute leukemia patients. Hypomethylating agents have been successfully used in AML patients with tuberculosis as a bridge to curative treatment, with 2 of 3 patients achieving complete remission in subsequent chemotherapy (20). However, some studies have reported cases where anti-tuberculosis treatment delayed the induction of remission of leukemia in various degrees or led to the abandonment of the conventional high-intensity chemotherapy regimens (21). Therefore, individualized treatment plans based on the patient’s specific condition are warranted in clinical practice.

Venetoclax is commonly used in chemotherapy for AML and associated with the most frequent adverse effects, including nausea, vomiting, and diarrhoea. Among severe adverse reactions, febrile neutropenia is the most frequently observed (22). A study observed that while the combination of azacitidine and venetoclax improves overall survival and remission rates compared to azacitidine monotherapy, it is also associated with a higher incidence of febrile neutropenia. The incidences of serious adverse events were 83% in the azacitidine-venetoclax group versus 73% in the monotherapy group (23). Another study, investigating patients treated with gilteritinib combined with azacitidine and venetoclax, found that the most common grade 3 or higher non-hematological adverse events were infection (62%) and febrile neutropenia (38%) (24). Previous literature has indicated that the adverse reactions to anti-tuberculosis drugs primarily include gastrointestinal reactions, hepatotoxicity, peripheral neuritis, ototoxicity, and hypersensitivity reactions (25). Therefore, regular monitoring of liver and kidney function should be performed during treatment, and timely intervention for adverse reactions is essential. In a few cases, isoniazid may affect white blood cell and platelet count and may even lead to severe myelosuppression (26) (27).

In the present case, the patient developed febrile neutropenia after chemotherapy. Initial clinical consideration focused on infections caused by common pathogens secondary to neutropenia, while tuberculosis was not initially suspected. A subsequent ultrasound of superficial lymph nodes throughout the body revealed retroperitoneal lymph node masses. The patient underwent the ultrasound-guided lymph node aspiration biopsy to clarify their nature. Pathological examination identified acid-fast bacilli, and the T-SPOT test returned positive, confirming the diagnosis of lymph node tuberculosis. The puncture site was the retroperitoneal lymph nodes, an anatomically complex area surrounded by blood vessels, which was associated with considerable technical difficulty and a relatively high risk of bleeding. Therefore, even with the ultrasound guidance, the procedure remained a technical challenge. Ultimately, the puncture biopsy was successfully performed, confirming the diagnosis of tuberculosis. For treatment, the original chemotherapy regimen of venetoclax combined with gilteritinib was continued. For drug interactions, given that rifampicin is a potent inducer of CYP3A4 (28) (29), which may reduce the plasma concentration of venetoclax, an anti-tuberculosis regimen consisting of isoniazid, ethambutol, and moxifloxacin was selected. Throughout treatment, the patient’s blood counts, liver function, and kidney function were monitored. No significant cytopenias or apparent abnormalities in liver or kidney function were observed, indicating a good tolerability. Ultimately, the patient achieved negative conversion on the T-SPOT test and maintained AML remission.

A history of tuberculosis, BCG vaccination, non-tuberculous mycobacterial infection, or immunodeficiency may all lead to false-positive results (30) (31). However, the patient’s related immune parameters, sputum culture, and blood culture results were all negative. The patient also had no history of BCG vaccination or tuberculosis infection. Additionally, tissue biopsy indicated tuberculosis, ruling out the possibility of a false positive. Ultimately, when the patient underwent a follow-up T-SPOT test, Ultimately, when the patient underwent a follow-up T-SPOT test, the symptoms of infection had resolved, the lymph node mase had a reduction, and leukemia was in remission, indicating effective treatment and demonstrating that the conversion to a negative T-SPOT result was clinically significant. The absence of the T-SPOT test before chemotherapy made it impossible to determine whether the patient’s tuberculosis resulted from reactivation of latent infection or from newly acquired infection during chemotherapy. This highlights that the T-SPOT test may be considered prior to AML chemotherapy to rule out latent tuberculosis infection. Mycobacterium tuberculosis infection occurring after AML chemotherapy is highly prone to misdiagnosis or underdiagnosis, particularly in the case of retroperitoneal lymph node tuberculosis. Once the retroperitoneal lymph node tuberculosis is suspected and no other evidence of tuberculosis is available, the ultrasound-guided puncture should be performed whenever feasible to clarify the pathological nature of the lymph nodes. Currently, there is no unified treatment standard for AML complicated with retroperitoneal lymph node tuberculosis. Individualized anti-tuberculosis regimens should be formulated promptly based on the patient’s condition, while simultaneously maintaining appropriate AML chemotherapy.

## Data Availability

The original contributions presented in the study are included in the article/supplementary material. Further inquiries can be directed to the corresponding author.
